# Spatial Modeling of Precipitation Based on Data-Driven Warping of Gaussian Processes

**DOI:** 10.3390/e24030321

**Published:** 2022-02-23

**Authors:** Vasiliki D. Agou, Andrew Pavlides, Dionissios T. Hristopulos

**Affiliations:** 1 School of Mineral Resources Engineering, Technical University of Crete, 73100 Chania, Crete, Greece; vagou@isc.tuc.gr (V.D.A.); apavlidis@isc.tuc.gr (A.P.); 2 School of Electrical and Computer Engineering, Technical University of Crete, 73100 Chania, Crete, Greece

**Keywords:** non-Gaussian data, skewed distributions, Gaussian anamorphosis, reanalysis data, kriging, warped Gaussian processes, 02.50.Fz, 02.60.Ed, 89.60.-k, 92.60.Ry, 05.10.Ln, 60G15, 60G60, 62F40, 62H11, 62G05, 65C05

## Abstract

Modeling and forecasting spatiotemporal patterns of precipitation is crucial for managing water resources and mitigating water-related hazards. Globally valid spatiotemporal models of precipitation are not available. This is due to the intermittent nature, non-Gaussian distribution, and complex geographical dependence of precipitation processes. Herein we propose a data-driven model of precipitation amount which employs a novel, data-driven (non-parametric) implementation of warped Gaussian processes. We investigate the proposed warped Gaussian process regression (wGPR) using (i) a synthetic test function contaminated with non-Gaussian noise and (ii) a reanalysis dataset of monthly precipitation from the Mediterranean island of Crete. Cross-validation analysis is used to establish the advantages of non-parametric warping for the interpolation of incomplete data. We conclude that wGPR equipped with the proposed data-driven warping provides enhanced flexibility and—at least for the cases studied– improved predictive accuracy for non-Gaussian data.

## 1. Introduction

Climate change combined with changes in land use is causing increased frequencies of drought and flooding events in many parts of the world. In 2021, extreme rainfall hit the Henan Province of China in July, Western Europe suffered severe flooding in mid-July, while extreme rainfall and flooding also affected the northern Amazon basin in South America and several parts of Africa. At the same time, prolonged droughts plagued several parts of the world. Such adverse impacts have been anticipated by scientists [1]. Climate and land-use changes affect ecosystems and human societies globally. One of the main concerns is their impact on the availability of water resources. It is thus important to better understand and forecast the spatial and temporal patterns of precipitation since these patterns affect the hydrological cycle and are crucial for the sustainability of human life on the planet.

Expert estimates—included in the Sixth Assessment Report of the Intergovernmental Panel on Climate Change—indicate an increase in the global averaged precipitation since 1950 [2]. In certain areas, both the frequency and intensity of heavy precipitation events have increased. In the Mediterranean region, on the other hand, it is expected that summer precipitation will decrease, thus increasing the risk of drought and aridification. In addition, there is evidence that the number of precipitation events has decreased while the intensity per event has increased [3]. Heavy precipitation events are expected to increase in several Mediterranean countries leading to increased flooding risks [4]. The impact of climate change on societies worldwide has renewed interest in quantitative methodologies that can estimate spatiotemporal climate and weather patterns [5]. Certain areas, including the Mediterranean basin, have been characterized as “climate change hot spots” [6]. Especially in such regions the interplay of climate change and changes in land use is crucial for water resource availability [4].

Accurate spatial models of precipitation are difficult to formulate, due to the variability and intermittent nature of precipitation across different temporal and spatial scales. In the Mediterranean region, the two large water bodies (the Atlantic Ocean and the Mediterranean Sea), as well as the major European mountain ranges are considered the main causes of extreme precipitation [7,8]. The total amount of precipitation received by an area over a specific time window is often modeled by means of parametric, non-Gaussian, probability distributions. If the temporal dimension is taken into account, modeling is further complicated due to the strong seasonal variability and intermittence of precipitation. Furthermore, despite significant progress over the last 30 years, the modeling of interactions between spatial and temporal correlations is an open research topic [9,10,11].

The spatial patterns of precipitation are calculated by means of stochastic spatial interpolation methods known as Kriging [12,13,14,15,16]. Kriging has been successfully used in environmental, meteorological, and hydrological studies to generate spatial maps based on partial data [17,18,19,20,21]. The predictive equations used by kriging also appear in the framework of Gaussian process regression (GPR) [22,23]. Kriging and GPR methods are based on the assumption of an underlying joint normal (Gaussian) distribution. However, the observed probability distributions of precipitation (as well as other environmental variables) are typically skewed (non-Gaussian) [24,25]. In geostatistical literature, non-Gaussian distributions are treated using nonlinear transformations that restore normality of the marginal distribution in a latent space; for reviews of such transformations see [14,16,23]. The application of nonlinear transformations to achieve normality is known as “Gaussian anamorphosis”. The spatial analysis is carried out in the latent space using the transformed data. Predictions in the observation space are derived by inverse transforming the predictions in the latent space. A similar “warping” approach has been applied to Gaussian process regression [26]. The term “warping” herein refers to the nonlinear transformation of the Gaussian process.

This study has two main objectives. First, we introduce a new, non-parametric (data-driven) warping approach. The warping transformation employs the kernel-based estimate of the cumulative distribution function (CDF) recently presented in [27]. This non-parametric method provides better estimates of the CDF of skewed probability distributions than other commonly used kernel-based methods. Secondly, we show that non-parametric warped Gaussian process regression (wGPR) can be used to model the spatial distribution of non-Gaussian variables such as precipitation. We focus on precipitation amounts because the respective probability distributions vary significantly in time, and the shape of the respective CDFs is not satisfactorily captured by parametric expressions. In order to assess the performance of *non-parametric wGPR*, we compare the results of cross-validation analysis with those obtained by non-warped GPR (i.e., Kriging). We apply cross-validation to a simulated noisy test function used in [26] as well as Leave-One-Out Cross-Validation (LOO-CV) to a reanalysis precipitation dataset from the island of Crete. The spatial dependence in the case of the test function is expressed in terms of a covariance model which corresponds to a linear, damped harmonic oscillator driven by white noise [23]. The enhanced interpolation accuracy provided by this model, which is not well-known in the machine learning and geostatistical literature, motivates its further use in Gaussian process regression.

The remainder of this paper is structured as follows: Section 2 presents the proposed wGPR methodology which involves Gaussian anamorphosis using the kernel-based CDF, spatial interpolation (prediction) of the normalized process employing standard GPR, and generation of the predictive distribution of precipitation by inverting the warping transformation. Section 3 presents an application of wGPR to a one-dimensional (1D) synthetic dataset which helps to illustrate the method. Section 4 compares GPR and wGPR using an ERA5 precipitation reanalysis dataset. Lastly, Section 5 presents discussion and conclusions and suggests future directions of research.

## 2. Methodology

Models of space-time processes rely on the spatial and temporal correlations inherent in the data. Most of the commonly used geostatistical approaches as well as GPR are developed to optimally perform Gaussian processes. However, non-Gaussian data are often encountered in nature. For example, precipitation amounts do not follow the Gaussian distribution. Typical models used include the exponential [28], gamma [29,30,31], lognormal [32,33,34], Weibull [28], generalized extreme value (GEV) [35,36,37,38,39,40], and Pareto distributions [41]. The optimal model depends on the geographical location, the climate zone, as well as the analyzed temporal and spatial scale [3,21,27]. It is thus necessary to relax Gaussian assumptions when modeling such data.

The wGPR approach proposed herein tackles non-Gaussian distributions using non-parametric warping of the observation space. The wGPR method involves the following steps: (i) Transformation of the data into standard normal values (normal scores) using a kernel-based warping function to conduct Gaussian anamorphosis; (ii) GP model specification based on variogram estimation; (iii) GPR using the normal scores; (iv) inversion of the warping transform to obtain predictions of precipitation values; and (v) calculation of cross-validation metrics for the assessment of predictive performance. These steps are described in more detail below.

### 2.1. Introduction to Gaussian Process Regression

A Gaussian process (GP) defines a prior distribution over functions which can then be used for Bayesian regression [22]. Herein we consider Gaussian processes having as input space the geographical coordinates $s\in D\subset R^{d}$ (d=1,2) of the domain of interest *D*. More generally, the input space can be extended to include the altitude and time as well as other potentially relevant for precipitation topographic parameters. Since a Gaussian process is fully determined by its mean and covariance kernel, we will denote the GP z(s) by means of $z\left(s\right)=\mathrm{GP}\left(m\left(s\right);C_{0}\left(s,s^{\prime }\right)\right)$, where $m\left(s\right):R^{d}\to R$ is the mean function (expectation) and $C_{0}\left(s,s^{\prime }\right):R^{d}\times R^{d}\to R$ is the covariance (kernel) function; the latter is a non-negative definite function. The mean function and the covariance kernel are determined from a set of *hyperparameters*. In geostatistical parlance, a Gaussian process whose input space is restricted to spatial location is a Gaussian random field [23].

Measurements of the process typically include a noise term ϵ(s) which represents a collection of independent identically (normally) distributed random variables with zero mean and constant variance $\sigma_{\epsilon }^{2}$. Then, the observed process is given by

(1) $$x\left(s\right)=z\left(s\right)+\epsilon \left(s\right),\;\mathrm{where}\;\epsilon ∼𝒩\left(0,\sigma_{\epsilon }^{2}\right),$$

𝒩(·,·) denotes the normal probability model, and the symbol ∼ implies that the probability distribution of the variable preceding ∼ follows the probability law specified after ∼. In the following, Φ(·) denotes the CDF of the standard normal distribution. The noise variance $\sigma_{\epsilon }^{2}$ is known in geostatistics as the nugget term [16].

Assuming that measurements are available at *N* points ${\left\{s_{i}\right\}}_{i=1}^{N}$, where $s_{i}\in D$, for all i=1,…,N, the joint PDF of the data is given by

(2) $$x{\left[x\left(s_{1}\right),\ldots ,x\left(s_{N}\right)\right]}^{⊤}∼\left(m_{s},C_{s}\right),$$

where ⊤ denotes the vector (matrix) transpose, $m_{s}={\left[m(s_{1},\ldots ,m\left(s_{N}\right)\right]}^{⊤}$ the vector of expected values, and ${\left[C_{s}\right]}_{i,j}={\left[C_{0}\right]}_{i,j}+\sigma_{\epsilon }^{2}\delta_{i,j}$, for i,j=1,…,N the elements of the covariance matrix.

Let us consider a set of points (in general this set is disjoint from the sampling set), $\left\{s_{1}^{*},\ldots ,s_{P}^{*}\right\}$ where the unknown values $z(s_{p}^{*})$, p=1,…,P of the process should be estimated. The joint PDF between the data and the predictions is given by

(3) $${\left[x,\;z\left(s_{1}^{*}\right),\ldots ,z\left(s_{P}^{*}\right)\right]}^{⊤}∼\left(\left[\begin{array}{c} m_{s}\\ m_{*} \end{array}\right],\left[\begin{array}{cc} C_{s} & C_{s,*}\\ C_{*,s} & C_{*,*} \end{array}\right]\right),$$

where $m_{*}={\left[m\left(s_{1}^{*}\right),\ldots ,m\left(s_{P}^{*}\right)\right]}^{⊤}$ is the vector of mean values, ${\left[C_{s,*}\right]}_{i,j}=C_{0}\left(s_{i},s_{j}^{*}\right)$ are the elements of the N×P covariance matrix between sampling and prediction points, $C_{*,s}=C_{s,*}^{⊤}$ is the P×N covariance matrix between the prediction and sampling points, and ${\left[C_{*,*}\right]}_{i,j}=C_{0}\left(s_{i}^{*},s_{j}^{*}\right)$ are the elements of the P×P covariance matrix between all pairs of the prediction points.

Since the joint PDF of the Gaussian process is normal, it is straightforward to obtain the conditional (on the data) PDF of the vector ${\left[x(s_{1},\ldots ,x\left(s_{N}\right)\right]}^{⊤}$ at the prediction points. The conditional PDF is also jointly normal with mean and covariance given respectively by the posterior mean, $m_{\mathrm{post}}$ and covariance, $C_{\mathrm{post}}$, i.e.,

(4) $${\left[z\left(s_{1}^{*}\right),\ldots ,z\left(s_{P}^{*}\right)\right]}^{⊤}|Data=𝒩(m_{\mathrm{post}},C_{\mathrm{post}}),$$

where the posterior mean is given by

(5) $$m_{\mathrm{post}}=m_{*}+C_{*,s}C_{s}^{-1}\;\left(x-m_{s}\right),$$

and the posterior covariance by means of

(6) $$C_{\mathrm{post}}=C_{*,*}-C_{*,s}C_{s}^{-1}\;C_{s,*}\;.$$

The GPR Equations (4)–(6) look identical to those of simple kriging [16]. However, in simple kriging, the mean is assumed constant and known, while in GPR the mean can comprise a superposition of basis functions with unknown coefficients (hyperparameters) which are estimated by maximizing the likelihood of the model [22]. Then, GPR is equivalent to universal kriging. More information regarding the relation between kriging and GPR can be found in [22,23]. Model selection in Gaussian processes is based on methods like Bayesian inference and cross-validation. In the former case, a prior function that captures *a priori* beliefs regarding the values of the hyperparameters is used.

Gaussian processes assume that the data follow the multivariate Gaussian distribution and that the observation noise is also Gaussian. The Gaussian assumption simplifies the calculations and leads to explicit predictive expressions. The optimal GPR prediction at a point $s_{p}^{*}$ is given by the conditional mean, $\hat{z}\left(s_{p}^{*}\right)=m_{\mathrm{post}}\left(s_{p}^{*}\right)$, while the uncertainty is determined by the conditional standard deviation $\hat{\sigma }\left(s_{p}^{*}\right)=\sqrt{C_{\mathrm{post}}\left(s_{p}^{*}\right)}$. The prediction interval at confidence level (1−α)×100% for 0<α<1 is given by

(7) $$\left[\hat{z}\left(s_{p}^{*}\right)-z_{\alpha /2}\;\hat{\sigma }\left(s_{p}^{*}\right),\;\hat{z}\left(s_{p}^{*}\right)+z_{1-\alpha /2}\;\hat{\sigma }\left(s_{p}^{*}\right)\right],$$

where $z_{\alpha /2}=\Phi^{-1}\left(\alpha /2\right)$ and $z_{1-\alpha /2}=\Phi^{-1}\left(1-\alpha /2\right)$ are, respectively, the (α/2)×100% and (1−α/2)×100% quantiles of the standard normal distribution.

### 2.2. Warping (Gaussian Anamorphosis) for Non-Gaussian Distributions

If the data follow a skewed probability distribution or exhibit heteroskedasticity (dependence of the variance on the spatial location), the assumption of normality may be inadequate. The standard approach for handling non-Gaussianity applies nonlinear normalizing transformations that restore marginal normality in a latent space. In geostatistical literature, this procedure is known as *Gaussian anamorphosis (GA)*. In the Gaussian process framework, *warped GPs* incorporate nonlinear “warping” transforms of the observation space [26].

Hence, “warping” applies a nonlinear transformation to a non-Gaussian GP $x\left(s\right)$, leading to a *latent Gaussian process* $y\left(s\right)$. More precisely, warping is defined as a *monotonic mapping* g:x↦y such that $y\left(s\right)=g\left[x\left(s\right)\right]$ has Gaussian marginal distribution with zero mean and unit variance [16,23]. Then, y(s) can be modeled as a Gaussian process—under the bold assumption that not only the marginal but also the joint PDF of any vector $y\in R^{N}$ is Gaussian.

If $F_{{\;}_{X}}\left(x\right)$ is the marginal CDF of $x\left(s\right)$, the *warping transform* g(·) is defined by means of g:x↦y such that

(8) $$y=\Phi^{-1}\left[F_{{\;}_{X}}\left(x\right)\right]=g\left(x\right)\;,$$

The inverse transformation from the latent to the observation space is given by the *inverse warping* function $\tilde{g}=g^{-1}\left(\cdot \right)$. The latter is defined by means of $\tilde{g}:y\mapsto x$ so that

(9) $$x=F_{{\;}_{X}}^{-1}\left[\Phi (y)\right]=\tilde{g}\left(y\right)\;$$

is given by the monotonic mapping from the latent Gaussian variable *y* to the observation variable *x* [14,23]. Often, closed-form expressions can be obtained for the functions g(·) and $\tilde{g}\left(\cdot \right)$. In other cases, they are numerically determined from the function composition of the CDF $F_{{\;}_{X}}\left(\cdot \right)$ with $\Phi^{-1}\left(\cdot \right)$ and of Φ(·) with the inverse $F_{{\;}_{X}}^{-1}\left(\cdot \right)$.

Predictions of y(s) in the warped space can be obtained by applying GPR as shown in Section 2.1. Transferring these predictions to the observation space is straightforward by means of the principle of *quantile invariance*, which states that the quantiles of a probability distribution remain invariant under a monotonic transformation, i.e., if $\Phi (y_{\alpha })=\alpha$ and $y_{\alpha }=g\left(x_{\alpha }\right)$, then it holds that $F_{{\;}_{X}}\left(x_{\alpha }\right)=\alpha$ [23]. Therefore, the predictive distribution in the observation space can be reconstructed from the respective distribution in the warped space by means of the function $\tilde{g}\left(y\right)$ defined in Equation (9). More precisely, the optimal prediction is given by

(10) $$\hat{z}\left(s_{p}^{*}\right)=\tilde{g}\left(\hat{y}\left(s_{p}^{*}\right)\right),$$

while the predictive interval at confidence level (1−α)×100% is given by

(11) $$\left[\tilde{g}\left(\hat{y}\left(s_{p}^{*}\right)-z_{\alpha /2}\;{\hat{\sigma }}_{y}\left(s_{p}^{*}\right)\right),\;\tilde{g}\left(\hat{y}\left(s_{p}^{*}\right)+z_{1-\alpha /2}\;{\hat{\sigma }}_{y}\left(s_{p}^{*}\right)\right)\right].$$

Note that Equation (10) returns the median of the marginal predictive distribution in the observation space. This is due to the principle of quantile invariance, taking into account that the conditional mean $\hat{y}\left(s_{p}^{*}\right)$ is also the median of the latent variable’s marginal conditional distribution.

### 2.3. Data-Driven Warping of Gaussian Processes

Non-parametric (data-driven) warping refers to model-free warping functions based on non-parametric estimates of the CDF from the data. Such estimates can be obtained using kernel functions and are more flexible than those provided by parametric models [27,42]. To avoid confusion, we emphasize that GPR is by construction a non-parametric method, in the sense that the underlying process is approximated without invoking a parametric model in the space of functions (albeit the GPs involve a number of hyper-parameters that control the shape of possible functions). So, the term “non-parametric” in relation to the wGPR refers to the data-driven warping function.

Closed-form parametric probability distributions are not sufficiently flexible to provide accurate models for the precipitation amount over different time scales. An example is the amount of precipitation in semi-arid Mediterranean areas, where the optimal model varies significantly across months but also across years for the same month [21]. In addition, parametric models that were accurate for past observations may not adequately capture future precipitation patterns due to climate change and the expected increase of extreme weather events [43]. Thus, the CDF of the data often cannot be accurately represented by a parametric model.

A non-parametric estimate of the CDF $F_{{\;}_{X}}\left(x\right)$ is obtained by integrating kernel density estimators (KDEs), leading to semi-explicit CDF expressions as shown in [27]. The method of kernel cumulative distribution function estimation (KCDE) uses an adaptive plug-in kernel bandwidth based on the theoretical considerations presented in [42]. The KCDE method is shown to provide better estimates of $F_{{\;}_{X}}\left(x\right)$ than the empirical (staircase) CDF estimate and kernel density estimation based on the normal-reference rule bandwidth.

A smoothing kernel is a real-valued, non-negative function $K\left(u;h\right)=K\left(\frac{u}{h}\right)$, which respects the properties of normalization, i.e., $\int_{-\infty }^{\infty }du\;K\left(u\right)=1$, and reflection symmetry, i.e., K(u)=K(−u). The parameter h>0 is the kernel bandwidth.

If $x_{\left[i\right]}$ represents the *i*-th order statistic (i.e., the *i*-th smallest value) of the sample vector x, the standard PDF kernel density estimator is given by [44]

(12) $${\hat{f}}_{K}\left(x\right)=\frac{1}{Nh}\sum_{i=1}^{N}K\left(\frac{x-x_{\left[i\right]}}{h}\right).$$

A kernel-based non-parametric estimate of the CDF $F_{{\;}_{X}}\left(x\right)$ can be obtained by means of the following weighted sum [27]

(13) $${\hat{F}}_{K}\left(x\right)=\sum_{i=1}^{N}\frac{1}{N}\;\tilde{K}\left(\frac{x-x_{\left[i\right]}}{h}\right).$$

In Equation (13), $\tilde{K}\left(\cdot \right)$ is the *CDF kernel step* defined by means of the following integral

(14) $$\tilde{K}\left(\frac{x-x_{\left[i\right]}}{h}\right)=\frac{1}{h}\int_{-\infty }^{x}dx^{\prime }\;K\left(\frac{x^{\prime }-x_{\left[i\right]}}{h}\right).$$

Equation (13) is obtained from Equation (12) using the integral ${\hat{F}}_{K}\left(x\right)=\int_{-\infty }^{x}{\hat{f}}_{K}\left(x^{\prime }\right)\;dx^{\prime }$. The CDF kernel steps are smoothed versions of the discontinuous steps used in the staircase CDF estimation. Explicit expressions of CDF kernel steps for various kernel functions are obtained in [27].

Once ${\hat{F}}_{K}\left(x\right)$ is known, it can be used in Equations (8) and (9) to obtain the warping transform and its inverse. Since it is not in general possible to derive explicit expressions for the warping function and its inverse, the function g(x) is defined in terms of a lookup table which contains CDF values, ${\left\{p_{i}\right\}}_{i=1}^{N_{d}}$, at $N_{d}=4\times 10^{3}$ discretization points; these are uniformly distributed over the interval $\left[x_{\min}-h,\;x_{\max}+h\right]$, where $x_{\min}$, $x_{\max}$ are respectively the minimum and maximum sample values and *h* is the kernel bandwidth. The respective values of g(x) are given by means of ${\left\{\Phi^{-1}\left(p_{i}\right)\right\}}_{i=1}^{N_{d}}$. The inverse transform requires finding *x* for a given probability level set by $p^{*}=\Phi \left(y^{*}\right)$. This is accomplished by linear interpolation of $x=F_{X}^{-1}\left(p\right)$ based on the two $p_{i}$ values in the lookup table nearest to $p^{*}$.

### 2.4. Hyperparameter Estimation

The spatial correlations of the process $x\left(s\right)$—or the transformed GP $y\left(s\right)$—are determined by means of the variogram function; the latter is also known as the second-order structure function in turbulence studies [45]. The variogram is defined as the semi-variance of the process’ increments, i.e., $\gamma_{Z}\left(r\right)=\frac{1}{2}Var\left[z\left(s+r\right)-z\left(s\right)\right]$ [16]. The variogram is purely a function of r if the increments are stationary (intrinsic hypothesis), a condition less strict than the stationarity of z(s).

In the case of stationary z(s), the covariance kernel and the variogram of the observed process x(s) are connected by means of the equation

(15) $$C\left(r\right)=\sigma^{2}-\gamma \left(r\right),$$

where $\sigma^{2}=\sigma_{\epsilon }^{2}+\sigma_{0}^{2}$ is the total variance and $\gamma \left(r\right)=\gamma_{Z}\left(r\right)+\sigma_{\epsilon }^{2}\left(1-\delta_{∥r∥,0}\right)$. As is known, the observation noise leads to a discontinuity of the variogram at the origin by $\sigma_{\epsilon }^{2}$ (nugget effect).

The variogram estimates are less sensitive to stochastic trends than covariance estimates due to the differencing operation implied in the increments. Thus, stochastic kriging methods are based on the variogram to generate spatial predictions [13,15,16]. An empirical variogram function can be straightforwardly estimated from the data using the *method of moments* [46]. The empirical variogram comprises a set of lag distances and respective estimates of the increment process’ semi-variance. This discrete function is then fitted to a theoretical model, i.e., a permissible (conditionally negative definite) function that is well-defined at every possible lag distance r. This procedure, albeit less efficient than maximum likelihood estimation, provides a computationally fast and visually clear alternative.

A list of commonly used variogram models is given in Appendix A.

### 2.5. Assessment of Predictive Performance

Cross-validation (CV) is a methodology that employs a set of statistical criteria in order to assess the predictive performance of spatial models. The data are split into two disjoint sets for training and validation. Strategies for selecting training and validation sets are reviewed in [47]. The training set is used to tune the GP hyperparameters. The validation data are used for comparison with the model predictions and assessment of the model’s performance [48]. A single training validation (100–400) split is used for the synthetic data example in Section 3.

A common strategy for sparse datasets is leave-one-out cross-validation (LOO-CV)—also known as delete-one CV [49], ordinary CV [50,51] or simply CV [52]. In LOO-CV the training set contains N−1 values and the validation set contains a single value. All *N* possible partitions of the data into training and validation sets are used. This approach is employed in Section 4 for assessing the GPR and wGPR performance on the precipitation reanalysis data.

The predictive performance of different models is assessed by means of statistical measures which include: the bias or mean error (ME), the mean absolute error (MAE), the root mean square error (RMSE), Pearson’s linear correlation coefficient (RP), the Nash-Sutcliffe coefficient (NS), the Empirical interval coverage (CVG), and the Negatively oriented interval score (NINTS) (see Appendix B for the definitions).

## 3. Application of GPR and Warped GPR to Synthetic Data

We apply the non-parametric wGPR approach to the synthetic 1D example used in [26]. The observation data are given by 100 random samples of

(16) $$x\left(s\right)={\left(\sin\left(\pi s\right)+\sigma_{\epsilon }\;\epsilon \left(s\right)\;\right)}^{1/3},\;s\in \left[-1,1\right],$$

where $\sigma_{\epsilon }=0.1$ and ϵ(s)∼N(0,1) provides the noise contamination of the underlying process $z\left(s\right)={\left(\sin(\pi s)\right)}^{1/3}$.

The process z(s) is approximated using GPR and wGPR. The performance of the reconstructions is evaluated by comparing the regression estimates with a sample ${\left\{z\left(s_{p}^{*}\right)\right\}}_{p=1}^{N_{v}}$ at $N_{v}=400$ uniformly distributed validation points in [−π,π]. In the case of GPR, the point predictions are obtained from the marginal posterior mean, Equation (5), the uncertainty is determined from the posterior covariance, Equation (6), and 95.45% prediction intervals are calculated according to Equation (7) with $z_{1-\alpha /2}=-z_{\alpha /2}=2$. In the case of wGPR, the prediction and the 95.45% predictive intervals are obtained from Equations (10) and (11) respectively.

**Figure 1:**
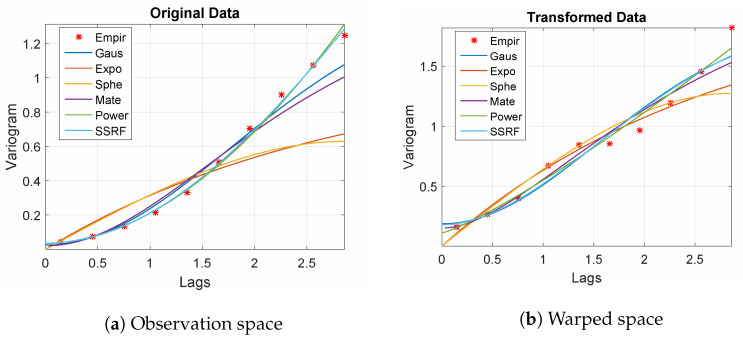
Empirical variograms (markers) and model fits (continuous lines) for the training set data (**a**) and the warped-space (normalized) data (**b**).

The empirical variograms for both the sample (in observation space) and its counterpart in the warped space are shown in Figure 1. Note that the spherical and exponential models, which imply continuous but non-differentiable stationary processes, provide poor fits for the empirical variogram (shown by markers), especially in the observation space. This is due to the fact that the true process is a differentiable function mixed with noise. All the other models tested provide reasonable fits to the empirical variograms (with the inclusion of a small discontinuity term at the origin which reflects the noise). Remarkably, the power-law model ($\gamma \left(r\right)=\alpha \;{\left|r\right|}^{2H}$) which corresponds to a non-stationary, continuous but non-differentiable process also gives a good fit. The best fit for the empirical variograms is provided by the Spartan model given by Equation (A4). In one dimension, the Spartan covariance model represents the covariance function of a damped linear harmonic oscillator driven by white noise [23], (Chapter 9). Hence, it is a suitable candidate for the oscillatory observation process defined by Equation (16).

The bitriangular kernel is used in wGPR for estimating the data-derived CDF based on which the warping is performed. The kernel is defined by means of $K\left(u\right)=\frac{3}{2}{\left(1-\left|u\right|\right)}^{2}$ for |u|≤1 and K(u)=0 for |u|>1. In both cases (GPR and wGPR) the Spartan variogram (covariance kernel) of Equation (A4) is used. Variograms corresponding to other covariance kernels (e.g., exponential, spherical, and Gaussian) were also studied. In general, non-differentiable kernels (e.g., spherical and exponential) lead to rougher reconstructions (exhibiting slope discontinuities) than the differentiable kernels (e.g., Gaussian and Spartan models).

Table 1 compares cross-validation metrics for the GPR and wGPR methods. Warped GPR achieves better performance in terms of most prediction metrics. High-performing prediction implies ME, MAE, and RMSE close to zero, RP and NS close to one, and small magnitudes of minimum and maximum errors. The bias (ME) is slightly lower for GPR, while the magnitudes of the minimum (ErrMin) and maximum (ErrMax) prediction errors are higher for wGPR than for GPR. The ErrMin and ErrMax values are influenced by the wGPR behavior near the left and right boundaries of the domain, where wGPR is closer than GPR to the observations. However, near the boundary the latter are mostly determined by ϵ(s) because z(s)≈0 near s=±π. The non-parametric warping transform used in wGPR is based on the CDF of the observations, thus enhancing adaptability to the data (for better or worse).

**Figure 2:**
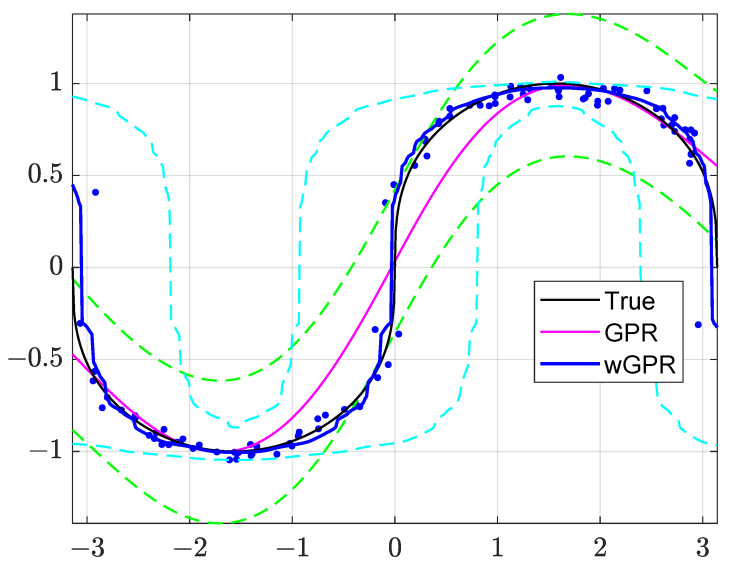
GPR and wGPR approximation of the function in Equation (16). Blue dots: Training set. Black line: The function z(s) plotted versus πs on the horizontal axis. GPR approximations (classical GPR: magenta line, warped GPR, blue line) and 95.45% prediction intervals (GPR: green dash lines, wGPR: cyan dash lines.)

The approximations of z(s) obtained by means of GPR and wGPR are illustrated in Figure 2: this figure shows the noisy training data (markers), the true process z(s) (black curve), as well as the GPR (magenta line) and wGPR (blue line) approximations with their respective 95.45% prediction intervals. Note that the validation values, $z(s_{p}^{*})$, lie on the black curve (unmarked). As evidenced in the plots of Figure 2, GPR provides a smooth, differentiable approximation of z(s), while wGPR yields a continuous but rough (non-differentiable) approximation that adapts more closely to the training data. In addition, the wGPR approximation is closer to the true z(s) almost everywhere except for the boundaries (as discussed above).

The GPR predictive intervals (green dashed lines) are symmetric around $\hat{z}\left(s\right)$, while the wGPR intervals (cyan dashed lines) are asymmetric and their width adjusts to the local slope of z(s). Both prediction intervals contain most of the test values $z(s_{p})$, p=1,…,400 (the prediction coverage is 100% for wGPR and ≈99% for GPR). On the other hand, GPR achieves this coverage with tighter intervals than wGPR. This is due to the fact that wGPR adapts the warping transform to the CDF of the training data, and is thus influenced by random errors.

## 4. Application of GPR and Warped GPR to Reanalysis Data

In this section, GPR and wGPR are applied to a sparse, non-Gaussian, precipitation reanalysis dataset.

### 4.1. Study Area and Data Description

The study area is the island of Crete (Greece) in the southeastern part of the Mediterranean basin. Crete is the largest island in Greece with an area of 8336 ${\mathrm{km}}^{2}$, length of 260 km, width ranging from 12 km to 57 km, and a maximum elevation of 2456 m. The island’s climate exhibits a transition from the Mediterranean to semi-arid as is common in Mediterranean regions [21,53]. Temperature and precipitation exhibit significant local variations due to three mountain ranges which are among the highest in Europe. The island is divided into four administrative regional units (from West to East): Chania, Rethymno, Heraklion, and Lasithi.

**Figure 3:**
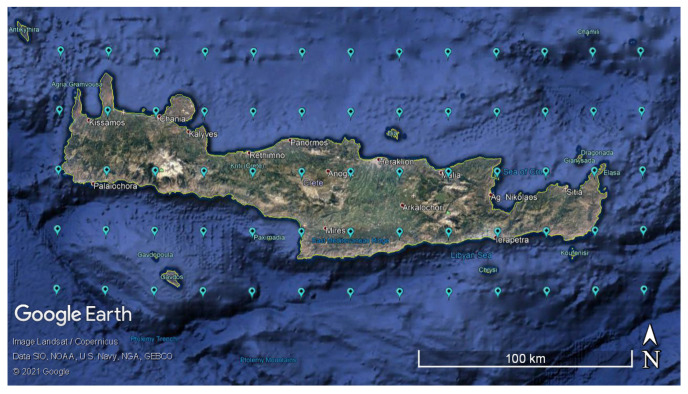
Geomorphological map of Crete showing the 65 nodes (blue markers) of ERA5 grid covering Crete, where the precipitation reanalysis data used in this study are located [59].

ERA5 reanalysis precipitation data were downloaded from the Copernicus Climate Change Service [54]. They include 23,360,610 values of hourly total precipitation for a period of 41 years (from 1 January 1979 06:00:00 to 31 December 2019 23:00:00) at the nodes of a 5×13 spatial grid (see Figure 3); the grid nodes are on and around the Greek island of Crete (see Figure 3). The average spatial resolution is ≈0.28 degrees (grid cell size ≈31 km). A total of 359,394 hourly precipitation values are available at every node.

Reanalysis is a systematic approach that employs data assimilation and numerical methods to generate weather and climate products over high-resolution grids [55]. Data assimilation involves mathematical techniques which can fuse data collected from several sources. Reanalysis products may contain bias due to errors and approximations in the observations and models used. Several studies have focused on the bias correction of meteorological variables, including precipitation, which are derived from reanalysis products [56,57,58]. This study does not apply bias correction methods since the aim is to validate the proposed wGPR methodology, not to compare reanalysis-based interpolation with results obtained from ground measurements.

To avoid modeling complications arising from zero values, we analyze monthly precipitation amounts for the wet season which involves the months from October until March. The resulting dataset includes 15,990 values of monthly precipitation amount (mm) for a period of 246 wet-season months (January 1979 to December 2019) at the 65 nodes of the ERA5 grid.

### 4.2. Exploratory Statistical Analysis

**Figure 4:**
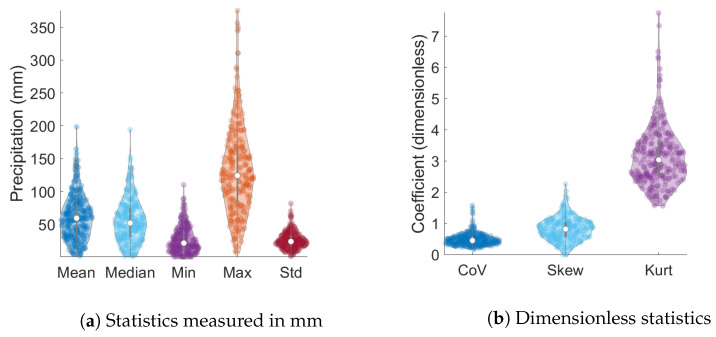
Violin plots for the mean, median, minimum and maximum values of monthly ERA5 precipitation statistics based on 246 monthly values. Each monthly statistic is based on the data at the 65 ERA5 grid nodes. The values for CoV (coefficient of variation), Skew (skewness) and Kurt (kurtosis) are dimensionless. All other values are measured in mm.

**Figure 5:**
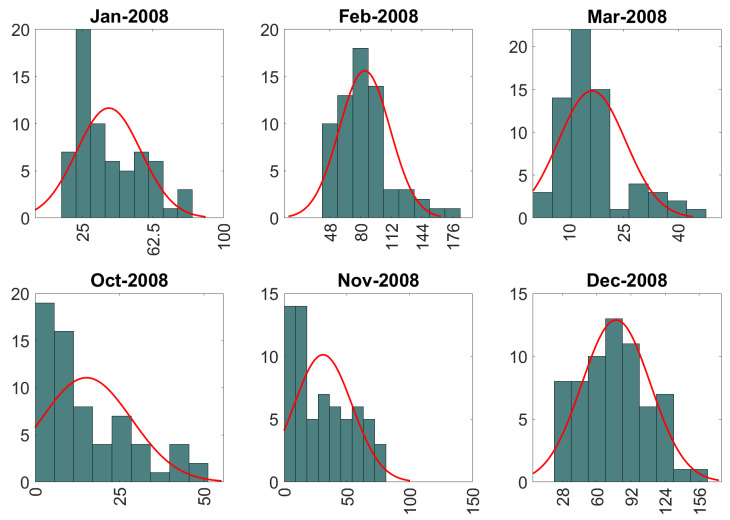
Distribution of monthly precipitation during the wet season of 2008. Histograms are based on ERA5 precipitation data at 65 grid locations over and around the island of Crete. Best fits to the optimal Gaussian PDF models (red line) are also shown. The vertical axis of the histograms represents frequency; the horizontal axis represents precipitation amount measured in mm.

Table 2 lists the summary statistics of the monthly precipitation data for the wet season. They include the mean value, the median, the minimum, and maximum values, the standard deviation, the coefficient of variation (ratio of the standard deviation over the mean), the skewness (coefficient of asymmetry), and the kurtosis. The way to read this table is as follows: the second column corresponds to the minimum value (evaluated over all months) of the monthly statistic shown along a given row (evaluated for each month from the 65 sites). The table is supplemented by Figure 4 which shows the probability distribution of the monthly statistics (corresponding to different columns of Table 2 calculated over the 246 months. These plots exhibit an asymmetric distribution of the statistics and considerable dispersion. The non-zero skewness, the deviation of the minimum and maximum kurtosis from the Gaussian value of three, and the unsuccessful fitting of the monthly histograms to the normal distribution (see Figure 5), strongly suggest that monthly precipitation data follow non-Gaussian distributions.

To investigate the deviations from Gaussian behavior the data are first grouped by location and then by month. The models that were tested include the generalized Pareto, inverse Gaussian, lognormal, t-Scale location, generalized extreme value, Weibull, Gaussian, Birnbaum-Saunders, exponential, extreme value, gamma, Nakagami, logistic, log-logistic, Rayleigh, and Rician probability distributions. According to Akaike’s Information Criterion (AIC), the Nakagami model is optimal at 45 of 65 nodes, the Weibull at 14, the gamma at 4, and the Rayleigh distribution at the remaining two locations. The results based on the Bayesian Information Criterion (BIC) are similar, with the Nakagami model being optimal at 40 of 65 nodes, the Weibull at 12, the gamma at 4, and the Rayleigh distribution at the remaining 9 nodes. For the data grouped by month, the optimal distribution according to AIC is the Nakagami model for 8 out of the 246 wet months, the Weibull for 3, the gamma for 13, the GEV for 25, the Rayleigh for 1, the generalized Pareto for 126, the log-logistic for 2, the lognormal for 3, the Birnbaum-Saunders for 25, and the inverse Gaussian distribution for the remaining 40 wet months.

In the analysis below, GPR and wGPR are applied to data for each wet-season month at all locations. Hence, a different spatial model is generated for each month. However, monthly data follow a different non-Gaussian probability distribution each month, and hence the warping function in wGPR changes every time. For illustration, the precipitation probability distributions for the year 2008 are investigated. Sixteen parametric probability distribution models (as listed above) were tested. The optimal probability model per each wet season month is presented in Table 3 (based on BIC) (see also Figure 5). The optimal distribution for most months is the generalized Pareto (GP). However, this only means that GP achieves a better BIC value than the other models, but it does not ensure that GP is an accurate representation of the empirical distribution.

### 4.3. GPR and Warped GPR Comparison Based on the Reanalysis Data

The following section presents LOO-CV analysis using GPR and wGPR for the six wet-season months over the 41-year period. The bitriangular kernel is used to estimate the non-parametric CDF which generates the warping function in wGPR. For both GPR and wGPR the spatial correlations are modeled by means of the exponential, Equation (A1), and Matérn, Equation (A3), variograms. The Spartan variogram, Equation (A5), was also tested, but the results obtained were nearly identical to those obtained with the exponential model. Hence, we kept the latter based on the principle of parsimony (the exponential model involves two hyperparameters compared to three for the Spartan model).

**Figure 6:**
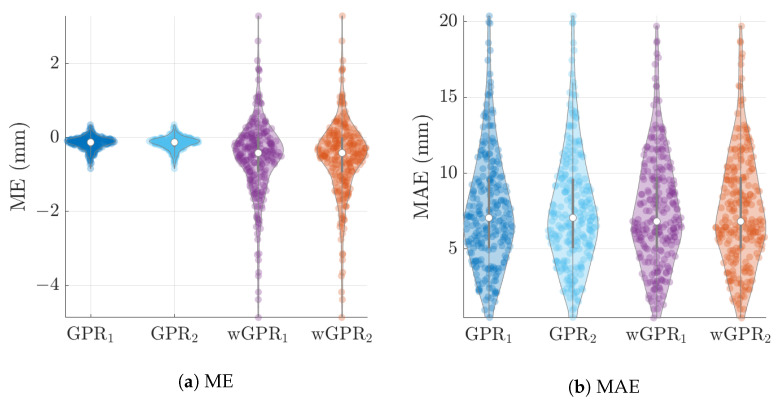
GPR and wGPR LOO-CV mean error (ME) and mean absolute error (MAE) for the wet-season ERA5 precipitation data. The lower indices “1” and “2” refer to the exponential and Matérn models respectively.

**Figure 7:**
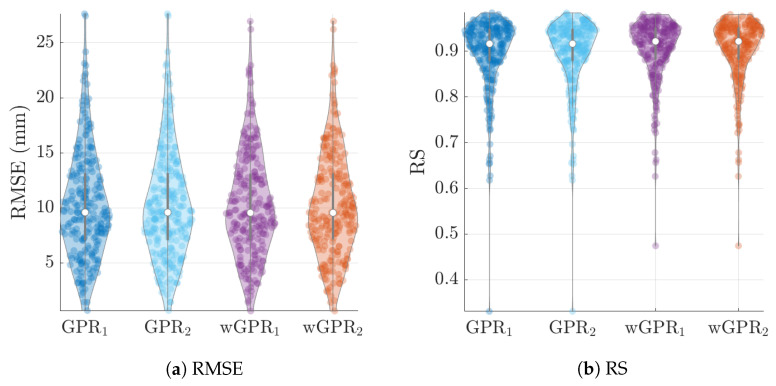
GPR and wGPR LOO-CV root mean error error (RMSE) and the Spearman correlation coefficient (RS) between the true and predicted values for the wet-season ERA5 precipitation data. The lower indices “1” and “2” refer to the exponential and Matérn models respectively.

**Figure 8:**
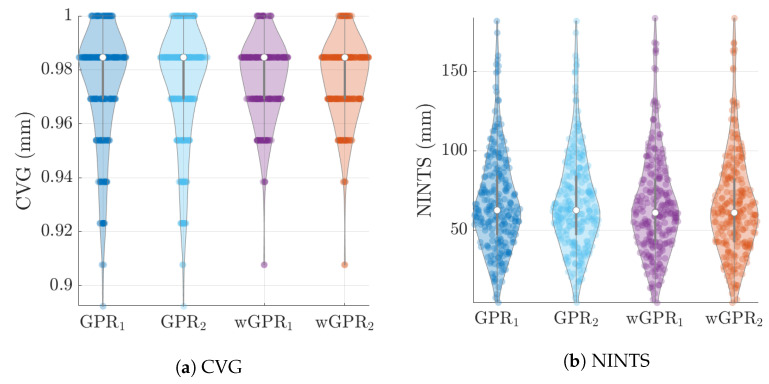
GPR and wGPR LOO-CV for two interval scores: the empirical interval coverage (CVG) and the negatively oriented interval score (NINTS) for the wet-season ERA5 precipitation data. The lower indices “1” and “2” refer to the exponential and Matérn covariance kernels respectively.

The distribution of LOO-CV metrics are shown in the violin plots of Figure 6, Figure 7 and Figure 8. Each violin plot is generated by 246 values (6 months × 41 years) of the respective LOO-CV metric which is calculated based on the 65 values at the grid nodes. The LOO-CV metrics used include the mean error (ME), the mean absolute error (MAE), the root mean square error (RMSE), and the Spearman correlation coefficient (RS). In addition to these, we also use two-interval scores (see Appendix B): the interval coverage (CVG) and the negatively oriented interval score (NINTS) [60]. Negatively oriented scores imply that lower scores correspond to better predictions.

The average values of the cross-validation metrics over all 246 time slices are shown in Table 4. The values of the metric have been rounded up to the second decimal place. Based on Table 4 and Figure 6, Figure 7 and Figure 8 there is practically no difference between the results obtained with the two different covariance kernels, in spite of the fact that the Matérn kernel allows for varying smoothness of the spatial function used to interpolate the data. The main differences are between GPR and wGPR. The mean error (bias) has a smaller magnitude for GPR than for wGPR. This is apparent in both the average values (−0.15 mm versus −0.53 mm) as well as the higher dispersion of the wGPR bias shown in Figure 6a. This behavior is expected since GPR is implemented so as to enforce a zero-bias constraint. Nonetheless, the wGPR bias is still a small fraction of the average minimum of the data (cf. Table 2). The wGPR-derived MAE and RMSE metrics are better than the respective GPR-based values, achieving both lower averages (Table 4) and lower extreme values (see Figure 6b and Figure 7a). The average values of the Spearman correlation are identical for both methods. However, as shown in Figure 7b, the wGPR lower tail of the Spearman correlation distribution is shorter than that of GPR; this implies that wGPR predictions do not lead to poor (rank) correlations with the true data. In terms of the interval scores, wGPR is superior since it leads to lower average NINTS and higher average coverage (CVG) values. In addition, as seen in Figure 8a the lower values of the wGPR interval coverage are higher than those of GPR.

## 5. Discussion and Conclusions

There is a strong interest in the application of Gaussian processes to model spatial and spatiotemporal data [61,62,63]. In the case of data exhibiting non-Gaussian distribution (e.g., precipitation), nonlinear transforms are applied to the observations in order to allow the application of Gaussian assumptions and methods. In geostatistics, this practice is known as Gaussian anamorphosis [14,16], while in machine learning this approach is known as Gaussian process warping [26,63]. In the study that introduced warped Gaussian processes, the hyperbolic tangent function is used to implement the warping transformation [26]. Other closed-form, nonlinear, monotonic transforms (e.g., square root, Box-Cox, logarithm, Tukey g-and-h) can be used for this purpose [23] (Chapter 14), [16,63,64].

In this study, we introduce a data-driven (non-parametric) warping method for Gaussian processes which employs kernel-based estimates of the data CDF. The latter is derived directly from the observations and conforms to the shape of the probability distribution that underlies the data. This is different from the Snelson et al. [26] approach which used an explicit warping function. To our knowledge the proposed method for warping Gaussian processes is new. The term “non-parametric warping” has also been used to denote deformations of the input space which can generate non-stationary Gaussian processes [61]. In this context, the warping function deforms the coordinate space so that in the new frame the resulting process can be considered stationary. In addition to applications in Gaussian processes, the non-parametric warping approach presented herein can also be used to perform Gaussian anamorphosis in the geostatistical framework.

Non-parametric warping of Gaussian processes allows greater flexibility than the use of parametric warping models: the shape of the data-driven warping function adapts to the features of the dataset at hand instead of being determined from a closed-form expression. Thus, non-parametric warping can provide improved approximation accuracy compared to GPR as evidenced in the 1D example studied in Section 3 and the precipitation reanalysis data (cf. Section 4). However, as shown in Section 3, non-parametric warping can lead to rough approximating functions if the data are contaminated by noise.

We combine non-parametric warping of Gaussian processes with Gaussian prediction (i.e., GPR/Kriging) and the principle of quantile invariance, in order to derive non-Gaussian marginal predictive distribution functions that capture the characteristics of the data distribution. The geostatistical method of variogram modeling is used to estimate the covariance kernel hyperparameters. Fitting the empirical variogram to theoretical models is a statistically less efficient procedure than likelihood maximization. However, it can lead to significant computational savings for large datasets where the $O(N^{3})$ computational cost of inverting the covariance (Gram) matrix may be prohibitive for likelihood optimization.

Our comparison of GPR and wGPR also employs monthly precipitation reanalysis (ERA5) data for the island of Crete. Reanalysis data provide valuable information regarding meteorological variables and the impact of climate change on their space-time patterns. Reanalysis data are especially useful in areas where environmental monitoring systems are sparse. As mentioned in Section 4, reanalysis datasets typically require bias correction in order to provide locally accurate estimates of precipitation. Bias correction can be conducted in combination with measurements from ground gauges, if such data are available. The GPR and wGPR methods can be used in combination with reanalysis and/or ground datasets to generate enhanced-resolution spatiotemporal maps for water resources management applications. A cross-validation comparison of the two methods (GPR and wGPR) showed that GPR has a lower bias, but wGPR is better with respect to other measures including interval scores. In addition, wGPR exhibits better performance in extreme cases where GPR led to poor cross-validation results. No significant differences are found between covariance kernels capable of exponential-like behavior (e.g., the exponential, Spartan, and Matérn models). This is attributed to the irregular spatial patterns of precipitation over Crete which are marked by exponentially decaying correlations [21].

The warped GPR model can be further investigated along the research directions described below. The present study does not model the dependence of precipitation on altitude. This can be accomplished by means of polynomial trend functions or other function bases. More complex measures of distance can be used in the covariance kernel to account for the anisotropic dependence of spatial precipitation patterns in the West-East and North-South directions of the island [21]. The scaling of the amount of precipitation with respect to the spatial support (i.e., the area over which the amount of precipitation is measured) is an important factor in the assessment of flood risk in ungauged watersheds [65], which needs to be linked to the interpolation procedure. The data from each wet-season month were herein treated as separate time slices. It is in principle possible to view the dataset in a space-time continuum and to adopt a spatiotemporal model for correlations. From the modeling perspective, the main difficulty is the construction of space-time covariance kernels that can adequately capture interactions between spatial and temporal correlations, as mentioned in the Introduction. From the numerical perspective, the covariance kernel inversion for the spatiotemporal problem has a significantly higher computation cost due to the respectively larger size of the dataset; the latter would in this case include $N=N_{t}\times N_{s}$ points, where $N_{s}$ is the number of ERA5 nodes and $N_{t}$ the total number of time slices. The issue of increased computational complexity can be addressed by replacing the Gaussian process with a stochastic local interaction model (SLI). The latter expresses space-time correlations in terms of sparse precision matrices. In SLI models the precision matrix is built using a data-adaptive strategy; this can lead to extremely sparse structures, thus reducing the computational cost of interpolation [23,66,67]. In addition to the above, in the space-time framework, a suitable temporal distance should be defined to capture seasonal trends in the precipitation patterns and space-time interaction of the correlations.

In conclusion, we have demonstrated that warped GPR equipped with non-parametric (data-driven) warping functions provides increased flexibility and enhanced accuracy for the spatial prediction of non-Gaussian distributions based on incomplete spatial datasets. The case studies that we examined include a synthetic test function with non-Gaussian noise and precipitation reanalysis data. The probability distributions representative of these datasets involves various degrees of departure from Gaussianity. Based on this evidence, we believe that wGPR will perform well in different applications that involve non-Gaussian data, although this remains to be explicitly demonstrated in future studies. Application of wGPR modeling may lead to poor results for certain types of non-Gaussian probability distributions: these include distributions that allow for isolated spatial extremes, and distributions with discrete spikes (e.g., zero-inflated precipitation during the dry season). The latter could be addressed by means of methods such as the variational, zero-inflated Gaussian process regression [68].

## Figures and Tables

**Table 1:** Cross-validation metrics for GPR and wGPR based on the validation set of 400 points from the function of Equation (16). ME: Mean error. MAE: Mean absolute error. RMSE: Root mean square error. RP: Pearson’s correlation coefficient. NS: Nash-Sutcliffe coefficient. ErrMin: $\min_{s_{1}^{*},\ldots ,s_{P}^{*}}\left(z\left(s_{p}^{*}\right)-\hat{z}\left(s_{p}^{*}\right)\right)$. ErrMax: $\max_{s_{1}^{*},\ldots ,s_{P}^{*}}\left(z\left(s_{p}^{*}\right)-\hat{z}\left(s_{p}^{*}\right)\right)$.

	ME	MAE	RMSE	RP	NS	ErrMin	ErrMax
GPR	−0.012	0.095	0.147	0.985	0.97	−0.50	0.38
wGPR	−0.016	0.050	0.119	0.990	0.98	−0.76	0.64

**Table 2:** Mean, median, minimum and maximum values (shown across rows) of monthly ERA5 precipitation statistics (shown across the columns) based on 246 monthly values (measured in mm). Each monthly statistic is based on the data at the 65 ERA5 grid nodes. The values for CoV (coefficient of variation), Skew (skewness) and Kurt (kurtosis) are dimensionless. All other values are measured in mm.

	Mean	Median	Min	Max	Std	CoV	Skew	Kurt
Mean	61.25	55.69	26.19	132.70	25.53	0.48	0.82	3.16
Median	59.19	51.78	21.23	123.98	23.67	0.45	0.81	3.04
Minimum	1.75	1.05	0.05	6.10	1.16	0.16	−0.01	1.56
Maximum	198.27	194.15	110.03	375.32	81.54	1.57	2.26	7.75

**Table 3:** Optimal probability distribution fits (based on BIC) for the monthly ERA5 precipitation data in the year 2008. The models studied include the following: “GP”: Generalized Pareto, “InvGauss”: Inverse Gaussian, “Logn”: Lognormal, and “Wei”: Weibull distribution. The optimal probability distributions for each wet-season month are not uniformly the same for different years.

January	February	March	October	November	December
GP	InvGauss	Logn	GP	GP	Wei

**Table 4:** Average values of LOO-CV metrics based on the 246 time slices of ERA5 precipitation data for the wet-season months.

	ME	MAE	RMSE	RS	NINTS	CVG
GPR (Expo)	−0.15	7.60	0.25	0.90	67.31	0.97
GPR (Mate)	−0.15	7.60	0.25	0.90	67.31	0.97
wGPR (Expo)	−0.53	7.53	0.21	0.90	65.50	0.98
wGPR (Mate)	−0.53	7.53	0.21	0.90	65.50	0.98

## Data Availability

The precipitation reanalysis datasets analyzed during the study are available in the ERA5-ECMWF dataset repository (ERA5|ECMWF). Restrictions apply to the availability of the data. Data was obtained from https://cds.climate.copernicus.eu/cdsapp#!/dataset/reanalysis-era5-land (accessed on 9 March 2020).

## Abbreviations

The following abbreviations are used in this manuscript:

AIC	Akaike Information Criterion
BIC	Bayesian Information Criterion
GEV	Generalized Extreme Value distribution
GP	Generalized Pareto distribution
GPR	Gaussian process regression
KCDE	Kernel cumulative distribution estimate
KDE	Kernel density estimate
wGPR	Warped Gaussian Process Regression

## Appendix A. Variogram Models

Variogram models define via Equation (15) the covariance kernel used in GPR. The models studied herein include the exponential, spherical, Matérn and Spartan models [23]. The relevant expressions are given below. In the following equations, ξ is the characteristic length of the spatial process.

Exponential model:

(A1) $$\gamma_{0}\left(r\right)=\sigma_{0}^{2}\;\left[1-\exp\left(-∥r∥/\xi \right)\right].$$

Spherical model:

(A2) $$\gamma_{0}\left(∥r∥\right)=\left\{\begin{array}{cc} \sigma_{0}^{2}\;\left[1.5\left(\frac{∥r∥}{\xi }\right)-0.5{\left(\frac{∥r∥}{\xi }\right)}^{3}\right], & \mathrm{if}\;∥r∥\leq \xi ,\\ \sigma_{0}^{2}\;, & \mathrm{if}\;∥r∥\geq \xi . \end{array}\right.$$

Matérn model:

(A3) $$\gamma_{0}\left(∥r∥\right)=\sigma_{0}^{2}\;\left[1-\frac{2^{1-\nu }}{\Gamma (\nu )}{\left(\sqrt{2\nu }\frac{∥r∥}{\xi }\right)}^{\nu }K_{\nu }\left(\sqrt{2\nu }\frac{∥r∥}{\xi }\right)\right].$$

For the Matérn model, ν>0 is the smoothness hyperparameter which controls the continuity of the process. Γ(·) is the gamma function, and $K_{\nu }\left(\cdot \right)$ is the modified Bessel function of the second kind of order ν.

Spartan model (*d* = 1):

(A4) $$\gamma_{0}\left(r\right)=\left\{\begin{array}{cc} \sigma_{0}^{2}-\eta_{0}e^{-\left|r\right|\beta_{2}/\xi }\left[\frac{\cos(\left|r\right|\beta_{1}/\xi )}{4\beta_{2}}+\frac{\sin(\left|r\right|\beta_{1}/\xi )}{4\beta_{1}}\right], & |\eta_{1}|<2,\\ \sigma_{0}^{2}-\eta_{0}\frac{1+|r|/\xi }{4}\;e^{-|r|/\xi },\; & \eta_{1}=2,\\ \sigma_{0}^{2}-\frac{\eta_{0}}{\sqrt{\eta_{1}^{2}-4}}\;\left[\frac{e^{-\left|r\right|\omega_{1}/\xi }}{2\omega_{1}}-\frac{e^{-\left|r\right|/\xi \omega_{2}}}{2\omega_{2}}\right], & \eta_{1}>2, \end{array}\right.$$

Spartan model (*d* = 2):

(A5) $$\gamma_{0}\left(∥r∥\right)=\left\{\begin{array}{cc} \sigma_{0}^{2}-\frac{\eta_{0}}{\pi \sqrt{4-\eta_{1}^{2}}}\;ℑ\left[K_{0}\left(\frac{∥r∥}{\xi \omega_{2}}\right)\right], & |\eta_{1}|<2,\\ \sigma_{0}^{2}-\frac{\eta_{0}∥r∥}{4\pi \xi }\;K_{-1}\left(\frac{∥r∥}{\xi }\right), & \eta_{1}=2,\\ \sigma_{0}^{2}-\frac{\eta_{0}}{2\pi \sqrt{\eta_{1}^{2}-4}}\;\left[K_{0}\left(\frac{∥r∥}{\xi \omega_{2}}\right)-K_{0}\left(\frac{∥r∥}{\xi \omega_{1}}\right)\right], & \eta_{1}>2\;. \end{array}\right.$$

For the Spartan model, the variance $\sigma_{0}^{2}$ is determined from the hyperparameters $\eta_{0}$, $\eta_{1}$ and ξ as follows [69]:

(A6) $$\left(d=1\right)\;\sigma_{0}^{2}=\left\{\begin{array}{cc} \frac{\eta_{0}}{2\sqrt{2+\eta_{1}}}, & |\eta_{1}|<2,\\ \frac{\eta_{0}}{4}, & \eta_{1}=2,\\ \frac{\eta_{0}}{2\sqrt{\eta_{1}^{2}-4}}\left(\omega_{1}^{-1}-\omega_{2}^{-1}\right),\; & \eta_{1}>2\;. \end{array}\right.$$

(A7) $$\left(d=2\right)\;\sigma_{0}^{2}=\left\{\begin{array}{cc} \frac{\eta_{0}}{2\pi \sqrt{4-\eta_{1}^{2}}}\left[\frac{\pi }{2}-\arctan\left(\frac{\eta_{1}}{\sqrt{4-\eta_{1}^{2}}}\right)\right], & |\eta_{1}|<2,\\ \frac{\eta_{0}}{4\pi }, & \eta_{1}=2,\\ \frac{\eta_{0}}{4\pi \sqrt{\eta_{1}^{2}-4}}\ln\left(\frac{\eta_{1}+\sqrt{\eta_{1}^{2}-4}}{\eta_{1}-\sqrt{\eta_{1}^{2}-4}}\right), & \eta_{1}>2\;. \end{array}\right.$$

In the Spartan model $\eta_{1}>-2$ is the rigidity hyperparameter (smaller $\eta_{1}$ allow oscillatory behavior of the covariance while $\eta_{1}\geq 2$ lead to exponential decay). The coefficients $\beta_{1,2}$ are determined as $\beta_{1,2}=\frac{1}{2}{\left|2\mp \eta_{1}\right|}^{1/2}$. The hyperparameters $\omega_{1,2}$ are dimensionless damping coefficients that determine the decay of the slow and fast exponential functions (for $\eta_{1}>2$). They are given by means of $\omega_{1,2}^{2}=\left(\eta_{1}\mp \frac{\sqrt{\eta_{1}^{2}-4}}{2}\right)$.

## Appendix B. Cross-Validation Metrics

The following equations define the leave-one-out cross-validation metrics used. The symbol ${\hat{z}}_{-i}\left(s_{i}\right)$ denotes the prediction at point $s_{i}\in R^{2}$ based on the N−1 data points excluding $s_{i}$; the true value at $s_{i}$ is represented by $z(s_{i})$; the spatial average of the data is denoted by $\bar{z(s_{i})}$; and $\bar{{\hat{z}}_{-i}\left(s_{i}\right)}$ is the spatial average of the predictions. The above metrics are straightforwardly extended to the case of disjoint training and validation sets.

Mean error (bias) (ME):

(A8) $$\varepsilon_{{\;}_{\mathrm{bias}}}=\frac{1}{N}\sum_{i=1}^{N}\left[z\left(s_{i}\right)-{\hat{z}}_{-i}\left(s_{i}\right)\right].$$

Mean absolute error (MAE):

(A9) $$\varepsilon_{{\;}_{\mathrm{MA}}}=\frac{1}{N}\sum_{i=1}^{N}\left|\;{\hat{z}}_{-i}\left(s_{i}\right)-z\left(s_{i}\right)\;\right|.$$

Root mean square error (RMSE):

(A10) $$\varepsilon_{{\;}_{\mathrm{RMS}}}=\sqrt{\frac{1}{N}\sum_{i=1}^{N}{\left[{\hat{z}}_{-i}\left(s_{i}\right)-z\left(s_{i}\right)\right]}^{2}}.$$

Pearson’s linear correlation coefficient (RP):

(A11) $${\bar{\rho }}_{P}=\frac{\mathrm{Cov}(z,\hat{z})}{\sigma_{z}\;\sigma_{\hat{z}}}=\frac{\sum_{i=1}^{N}\left[z\left(s_{i}\right)-\bar{z(s_{i})}\right]\left[{\hat{z}}_{-i}\left(s_{i}\right)-\bar{{\hat{z}}_{-i}\left(s_{i}\right)}\right]}{\sqrt{\sum_{i=1}^{N}{\left[z\left(s_{i}\right)-\bar{z(s_{i})}\right]}^{2}}\sqrt{\sum_{i=1}^{N}{\left[{\hat{z}}_{-i}\left(s_{i}\right)-\bar{{\hat{z}}_{-i}\left(s_{i}\right)}\right]}^{2}}}.$$

Spearman’s rank correlation coefficient is also obtained from Equation (A11) by replacing $z,\hat{z}$ with $R\left(z\right),R\left(\hat{z}\right)$, where R(·) represents the rank function.

Nash-Sutcliffe coefficient (NS):

(A12) $$\varepsilon_{{\;}_{\mathrm{NS}}}=1-\frac{\sum_{i=1}^{N}{\left[{\hat{z}}_{-i}\left(s_{i}\right)-z\left(s_{i}\right)\right]}^{2}}{\sum_{i=1}^{N}{\left[z\left(s_{i}\right)-\bar{z(s_{i})}\right]}^{2}}.$$

Empirical interval coverage (CVG):

(A13) $$\begin{array}{cc} \mathrm{CVG}= & \frac{1}{N}\sum_{i=1}^{N}{\mathrm{CVG}}_{i}, \end{array}$$

(A14) $$\begin{array}{cc} {\mathrm{CVG}}_{i}= & 𝟙\left(z\left(s_{i}\right)>l_{i}\wedge z\left(s_{i}\right)<u_{i}\right) \end{array}$$

In the above, 𝟙(·) is the indicator function: 𝟙(A)=1 if *A* is true and 𝟙(A)=0 if *A* is false. In addition, $l_{i}$ and $u_{i}$ represent, respectively, the α/2 and 1−α/2 quantiles of the predictive distribution at the point $s_{i}$. The CVG thus represents the fraction of points where the prediction interval (at a specified quantile level 0<α<1) contains the true value of the sample. Herein it is assumed that α=4.55%.

Negatively oriented interval score (NINTS):

(A15) $$\begin{array}{cc} {\bar{S}}_{\alpha }= & \frac{1}{N}\sum_{i=1}^{N}S_{{\;}_{\alpha }}\left(l_{i},u_{i},z\left(s_{i}\right)\right), \end{array}$$

(A16) $$\begin{array}{cc} S_{{\;}_{\alpha }}\left(l,u,x\right)= & \left(u-l\right)+\frac{2}{\alpha }\left(l-x\right)𝟙\left(x<l\right)+\frac{2}{\alpha }\left(x-u\right)𝟙\left(x>u\right). \end{array}$$

The $l_{i}$ and $u_{i}$ are defined above for the CVG.
